# Gender-specific pathways to leadership competency: the role of emotional intelligence and self-esteem among Saudi nursing students

**DOI:** 10.3389/fmed.2025.1744198

**Published:** 2026-01-12

**Authors:** Fathia Ahmed Mersal, Ibrahim Naif Alenezi

**Affiliations:** Public Health Nursing, College of Nursing, Northern Border University, Arar, Saudi Arabia

**Keywords:** emotional intelligence, gender differences, leadership competency, nursing students, Saudi Arabia, self-esteem, Vision 2030, Wong and Law Emotional Intelligence Scale

## Abstract

**Background:**

Leadership competency is vital in nursing education to advance Saudi Arabia’s Vision 2030 healthcare reforms. Emotional intelligence (EI) and self-esteem (SE) are known predictors of leadership, yet their gender-specific influence among Saudi nursing students remains unclear.

**Purpose:**

This study examined how EI and SE predict leadership competency among male and female Saudi nursing students, guided by Gender Role Theory, Trait Leadership Theory, Granular Interaction Thinking Theory, and the Bayesian Mindsponge Framework.

**Methods:**

A comparative cross-sectional design included 260 nursing students from Northern Border University. Participants completed the Leadership Competency Scale (40 items; *α* = 0.91), Wong and Law Emotional Intelligence Scale (16 items; *α* = 0.88), and Rosenberg Self-Esteem Scale (10 items; *α* = 0.82). Confirmatory factor analysis validated all instruments. Data were analyzed using Mann–Whitney *U* tests and hierarchical multiple regression with moderation analysis.

**Results:**

Males reported higher leadership competency (*M* = 139.33, SD = 30.45) than females (*M* = 94.89, SD = 32.14; *p* < 0.001, *d* = 1.42). Conversely, females scored higher in EI (*M* = 95.39, SD = 23.04) than males (*M* = 67.45, SD = 25.75; *p* < 0.001, *d* = −1.14). This paradox suggests EI does not translate into leadership equivalently across genders. Gender-stratified regressions revealed distinct pathways: for males, EI predicted leadership (*β* = 0.28, *p* = 0.004), with academic year (*β* = 0.275, *p* = 0.005) and age (*β* = 0.223, *p* = 0.023) as contributors, while SE acted as a suppressor (*β* = −0.315, *p* < 0.001), explaining 22.9% of variance. For females, SE was the strongest predictor (*β* = 0.293, *p* = 0.001) alongside academic year (*β* = 0.244, *p* = 0.007), while EI showed no predictive utility (*β* = −0.037, *p* = 0.63), accounting for 29.9% of variance. A significant EI × Gender interaction (*β* = −0.37, *p* = 0.020) confirmed differential patterns.

**Conclusion:**

Leadership competency among Saudi nursing students follows gender-specific pathways. For males, EI enhances leadership, while for females, SE is the key predictor. Educational strategies should prioritize EI development for males and SE for females to achieve equitable leadership outcomes aligned with Vision 2030.

## 1 Background

Leadership development among nursing students represents a critical priority in contemporary healthcare education, particularly as healthcare systems grow increasingly complex and demand adaptive, emotionally intelligent leaders. Nursing leadership competencies, including effective communication, self-awareness, emotional regulation, and strategic decision-making, must be systematically cultivated during undergraduate education to ensure graduates’ readiness for complex clinical environments (1). This imperative is especially pronounced in Saudi Arabia, where the national Vision 2030 framework explicitly prioritizes healthcare transformation and nursing leadership as fundamental pillars of sustainable development and positions nurse leaders as key agents in achieving quality care delivery, patient safety improvements, and workforce empowerment (2, 3). Vision 2030’s ambitious healthcare transformation goals, including achieving universal health coverage, improving patient outcomes, reducing healthcare-associated infections, and establishing Saudi Arabia as a regional healthcare leader, necessitate a robust pipeline of nurse leaders equipped with both technical competencies and sophisticated psychological capabilities (3).

However, the cultivation of nursing leadership must commence during undergraduate education rather than postponing development until professional practice. Nursing students represent the foundational cohort whose leadership competencies will ultimately determine the success of healthcare transformation initiatives. The developmental trajectories, psychological mechanisms, and competency outcomes differ fundamentally between nursing students and practicing nurses due to distinct contextual demands, experiential learning opportunities, and identity formation processes (4, 5). Professional nurses operate within established clinical hierarchies, possess accumulated practical experience, and navigate complex organizational politics, factors that moderate how emotional intelligence and self-esteem translate into leadership behaviors. Conversely, nursing students are in formative developmental stages where psychological attributes such as emotional intelligence and self-esteem serve as foundational resources for constructing leadership identities, navigating educational challenges, and preparing for professional role transitions (1). Therefore, examining leadership determinants specifically among nursing students provides critical insights into early-stage developmental mechanisms that educational interventions can effectively target, thereby ensuring that graduates enter the workforce with established leadership capabilities aligned with Vision 2030’s transformation agenda.

Central to this development is EI, which Coronado-Maldonado and Benítez-Márquez (6) identify as a foundational attribute that fosters empathy, resilience, and team cohesion while reducing burnout and improving patient care quality. Emotional intelligence encompasses the capacity to recognize, understand, and regulate one’s own emotions while simultaneously perceiving and influencing others’ emotional states, a multidimensional competency increasingly recognized as indispensable for effective healthcare leadership (7). Recent empirical evidence demonstrates that EI significantly predicts leadership effectiveness, team performance, and organizational commitment within healthcare contexts (8). Furthermore, integrating EI development with reflective practice in nursing curricula has been shown to enhance students’ leadership readiness, critical thinking, and decision-making capabilities under conditions of clinical uncertainty (9).

Systematic reviews and meta-analyses demonstrate that nurses with higher emotional intelligence exhibit superior communication skills, enhanced empathy, and greater resilience when managing workplace stress and interpersonal conflicts (7). These capabilities translate into tangible organizational benefits, including reduced staff turnover, improved job satisfaction, and enhanced patient care quality. Qualitative investigations employing hermeneutic phenomenological approaches revealed that emotionally intelligent nursing care fosters deeper patient connections and more adaptive clinical responses (10). Additionally, research conducted specifically within Saudi Arabian healthcare contexts found that nurses with higher emotional intelligence demonstrated stronger interpersonal relationships and leadership potential, reinforcing EI’s role in cultivating transformational leadership behaviors within culturally diverse healthcare environments (11).

Complementing emotional intelligence, self-esteem emerges as another crucial psychological determinant of leadership development. Self-esteem, representing an individual’s overall evaluation of their worth and competence, influences confidence in decision-making, willingness to assume leadership responsibilities, and resilience when facing challenges (12). In nursing contexts characterized by high-stakes clinical situations, hierarchical organizational structures, and frequent exposure to professional scrutiny, self-esteem functions as a psychological foundation that enables individuals to engage confidently in leadership behaviors, advocate for patients and colleagues, and persist through adversity (13). Empirical evidence suggests that self-esteem operates not only as a stable trait but also as a dynamic psychological resource that interacts with situational demands and social contexts to shape leadership capacity (14).

Despite growing recognition of emotional intelligence and self-esteem as important leadership determinants, significant gaps remain in understanding how these psychological factors interact with gender to influence leadership development. While some studies suggest universal benefits of emotional intelligence across healthcare professions, emerging evidence indicates that the mechanisms through which psychological traits translate into leadership competency may differ fundamentally between male and female nursing students. Research examining gender and generational differences in emotional intelligence and transformational leadership found that gender-specific socialization patterns influence how emotional competencies are expressed and perceived in leadership roles (15), aligning with gender role theory, which posits that societal norms, cultural expectations, and differential socialization experiences shape behavioral tendencies, self-perceptions, and leadership expression differently across genders (16, 17). Gender role theory specifically articulates that males and females internalize distinct normative expectations regarding appropriate behaviors, emotional expression patterns, and role responsibilities, socialization processes that fundamentally shape how psychological attributes such as emotional intelligence and self-esteem are developed, expressed, and leveraged in leadership contexts (17).

In Middle Eastern contexts, these gender dynamics become particularly complex due to the intersection of traditional cultural norms with contemporary healthcare transformation initiatives. Saudi Arabia’s healthcare system is undergoing unprecedented structural, professional, and cultural transformation, with explicit national policies promoting female workforce participation, leadership development, and professional advancement as core strategic objectives (2). However, nursing in Saudi Arabia operates within unique cultural parameters where traditional gender role expectations, religious values, and evolving societal norms create a complex sociocultural environment that may significantly moderate how emotional intelligence and self-esteem contribute to leadership development trajectories among male and female students (18).

The limited research examining gender-specific pathways to leadership development in nursing education represents a critical knowledge gap. While international studies have established the importance of emotional intelligence and self-esteem for nursing leadership broadly, findings derived predominantly from Western, individualistic cultural contexts may not generalize directly to collectivistic Middle Eastern settings where different cultural values, gender norms, family structures, and organizational hierarchies fundamentally moderate relationships between psychological traits and leadership outcomes (19). Furthermore, most existing research has been conducted in Western settings, limiting generalizability to Middle Eastern healthcare contexts where different cultural values and organizational structures may moderate relationships between psychological traits and leadership outcomes.

This knowledge gap carries substantial implications for Saudi Arabia’s healthcare transformation strategy. Understanding the gender-specific psychological mechanisms that facilitate or constrain leadership competency development becomes essential for designing evidence-based educational interventions that optimize leadership preparation for all students while respecting cultural contexts. Without this understanding, nursing education programs risk perpetuating gender disparities in leadership development, failing to leverage the unique psychological strengths that male and female students bring to leadership roles, or implementing generic interventions that prove ineffective when gender-differentiated developmental pathways exist.

The present study seeks to address a critical gap in nursing education research by exploring the interplay between emotional intelligence, self-esteem, and leadership competency within the context of gender differences among nursing students in Saudi Arabia. Specifically, it aims to examine the differential predictive relationships between emotional intelligence and leadership competency for male and female students, as well as the corresponding relationships between self-esteem and leadership competency. Furthermore, the study investigates whether gender moderates the relative importance of emotional intelligence versus self-esteem in predicting leadership competency, thereby offering nuanced insights into gender-specific dynamics. Ultimately, this research endeavors to provide empirical evidence that can inform the design of gender-responsive educational interventions for nursing leadership development, aligning with the transformative goals of Saudi Arabia’s Vision 2030 healthcare agenda.

### 1.1 Theoretical integration and conceptual framework

**Figure 1:**
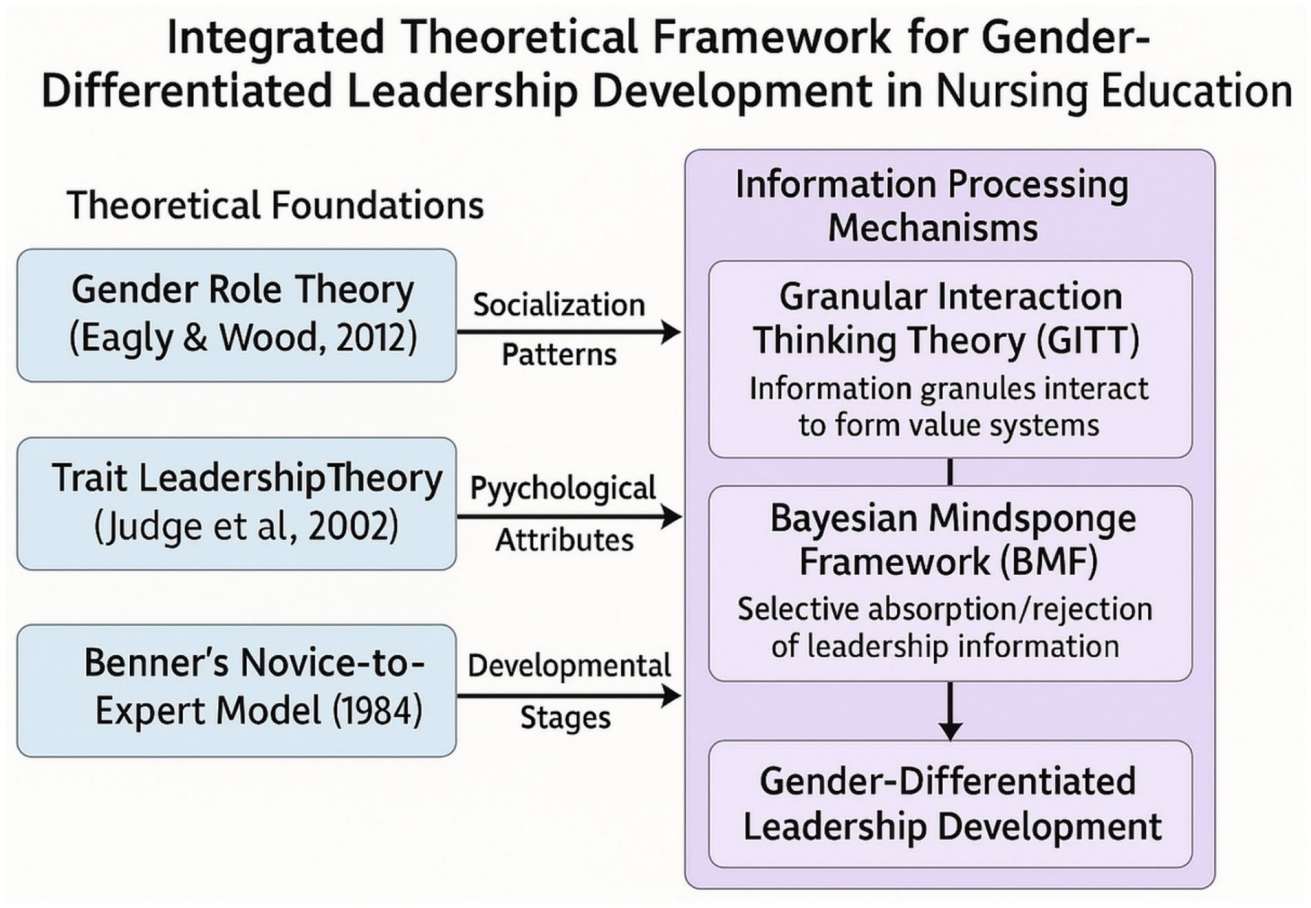
Integrated theoretical framework for gender-differentiated leadership development in nursing education.

This study adopts an integrative framework combining trait-based, sociocultural, developmental, and information-processing perspectives to explain gendered pathways to leadership among nursing students. Emotional intelligence and self-esteem remain central predictors of leadership potential (7, 20, 21). Gender role theory emphasizes how social norms and differential socialization shape trait expression and leadership behaviors (22, 23). Nursing leadership development is further contextualized within Saudi Arabia’s Vision 2030, which prioritizes strategic leadership capacity building (3, 24). Contemporary frameworks such as the Granular Interaction Thinking Theory and Bayesian Mindsponge Framework provide cognitive models explaining how individuals selectively absorb or reject leadership-related information based on value alignment and cost–benefit evaluations (25). Integrating these perspectives, we hypothesize that female students, socialized toward communal values, may rely more on self-esteem, whereas male students in a female-typed profession may actively cultivate EI to resolve role incongruity. Academic progression may further moderate these dynamics, as transitions between stages trigger cognitive restructuring through mindsponge mechanisms. This cross-sectional study examines differential predictive relationships between EI, self-esteem, and leadership competency among male and female nursing students in Saudi Arabia, aiming to inform gender-responsive interventions aligned with Vision 2030 healthcare transformation goals (Figure 1).

This conceptual framework integrates multiple theoretical perspectives to elucidate how EI and SE predict leadership competency among nursing students in Saudi Arabia. Grounded in Gender Role Theory, Trait Leadership Theory, and Benner’s Novice-to-Expert Model, it situates leadership development within socialization patterns, psychological attributes, and professional progression stages. Cognitive mechanisms, informed by Granular Interaction Thinking Theory and the Bayesian Mindsponge Framework, explain how leadership-relevant information is selectively processed to form value systems, producing gender-differentiated outcomes. Gender moderates the predictive strength of EI and SE, while contextual factors, such as Vision 2030 reforms, cultural dynamics, and academic progression, shape these associations. Although the cross-sectional design limits causal inference, this framework provides a robust theoretical foundation for examining psychological predictors of leadership within the evolving Saudi healthcare context.

## 2 Materials and methods

### 2.1 Study design and setting

A comparative cross-sectional design was employed to examine associations between EI, SE, and leadership competency among nursing students at Northern Border University in Saudi Arabia. This design enabled simultaneous measurement of multiple psychological constructs and identification of gender-based patterns at a single time point, though it precludes causal inference due to the absence of temporal precedence (26). The study was conducted within NBU’s College of Nursing during the 2024–2025 academic year from September 2024 to March 2025. Data collection took place in dedicated classroom spaces to ensure standardized conditions and minimize environmental variability. NBU serves as a representative institution within Saudi Arabia’s Northern Border Region, providing a culturally relevant context for examining gender dynamics in nursing leadership development aligned with Vision 2030 healthcare transformation initiatives. However, findings from this single-institution study may have limited generalizability to other Saudi nursing programs or international contexts with different cultural, educational, or healthcare system characteristics.

To minimize potential confounding factors such as cultural norms, family influences, and gender segregation, all participants were recruited from the same nursing college at Northern Border University. They shared the same curriculum, faculty, and clinical training environment within the same region of Saudi Arabia under the Vision 2030 policy framework. This institutional and cultural homogeneity reduces variability and strengthens internal validity.

### 2.2 Participants

Census sampling was employed to recruit all accessible undergraduate nursing students enrolled in the second, third, and fourth academic years (*N* = 260). This approach was justified by the manageable population size and the study’s aim to capture comprehensive gender representation within the institution. Eligibility criteria included active enrollment in the nursing program, a minimum age of 18 years, and proficiency in both Arabic and English to ensure comprehension of all study instruments administered in their validated language versions. Students on extended leave or with documented cognitive impairments affecting survey completion were excluded. The sample achieved gender balance (128 male, 132 female students), enabling robust comparative analyses. Notably, participants’ exposure to formal leadership education varied according to their academic progression, with fourth-year students having completed dedicated leadership and management courses while second and third-year students were still developing foundational competencies. This variation aligns with Benner’s (27) novice-to-expert framework, which posits that clinical and professional competencies develop progressively through educational experiences and practice exposure.

Recruitment proceeded through a gender-stratified protocol. The research team obtained official permission from NBU’s College of Nursing administration, followed by separate information sessions conducted for male and female cohorts to explain study purposes and procedures while maintaining cultural appropriateness. To mitigate social desirability bias arising from potential power dynamics between student participants and university-affiliated researchers, several protective measures were implemented: (1) complete anonymity was ensured through non-traceable electronic submissions without personal identifiers, (2) participants were explicitly informed that responses would not be accessible to course instructors or affect academic evaluation, and (3) aggregate reporting protocols prevented individual response identification. Additionally, the survey was distributed via a secure Google Form link shared with student leaders, who then disseminated the link to their peers, ensuring voluntary participation and minimizing perceived institutional influence.

Unique Google Forms links were distributed to each gender cohort via NBU’s official learning management system. Digital informed consent was obtained through a mandatory preliminary question in Google Forms, requiring agreement before proceeding. Participants completed the electronic questionnaire independently over 7 days, with reminder emails sent at 48-h intervals to non-respondents.

### 2.3 Measurements

Data collection utilized a structured questionnaire comprising four sections: demographic information, self-esteem assessment, emotional intelligence assessment, and leadership competency evaluation. All instruments were administered in their validated English and Arabic versions to ensure linguistic consistency and cultural appropriateness for the predominantly Arabic-speaking student population. This approach was particularly relevant as nursing programs in Saudi Arabia are delivered primarily in English, ensuring that participants were familiar with the language of instruction while maintaining accessibility through Arabic translations.

#### 2.3.1 Demographic information

Participants provided age, gender, academic year, and grade point average (GPA) through closed-ended items. GPA was collected as a proxy measure of academic achievement to examine potential associations between scholastic performance and leadership competency development, based on theoretical frameworks suggesting that cognitive abilities and academic success may contribute to leadership capacity (28). This variable enabled exploratory analyses of whether academic excellence correlates with self-reported leadership behaviors in the nursing education context.

#### 2.3.2 Rosenberg Self-Esteem Scale

The validated Arabic version of the Rosenberg Self-Esteem Scale (RSES; (29)) measures self-esteem through 10 items rated on a 4-point Likert scale ranging from 1 (Strongly Disagree) to 4 (Strongly Agree). Five items (2, 5, 6, 8, 9) were reverse-scored. Total scores range from 10 to 40, with higher scores indicating greater self-esteem. Example items include “I feel that I have a number of good qualities” and “I am able to do things as well as most other people.” The RSES has demonstrated strong psychometric properties across diverse cultural contexts and showed excellent internal consistency in the current sample (Cronbach’s *α* = 0.82).

#### 2.3.3 Wong and Law Emotional Intelligence Scale

The Wong and Law Emotional Intelligence Scale [WLEIS; (30)] was administered in both English and Arabic versions to ensure linguistic and cultural appropriateness for the predominantly Arabic-speaking student population, while maintaining consistency with the English-based nursing curriculum. The Arabic version was developed through a rigorous forward–backward translation process and reviewed by bilingual experts for semantic equivalence. To confirm linguistic accuracy and conceptual clarity, a pilot test was conducted with a sample of bilingual nursing students.

The WLEIS assessed EI across four domains: Self-Emotions Appraisal (SEA, 4 items), Others’ Emotions Appraisal (OEA, 4 items), Use of Emotion (UOE, 4 items), and Regulation of Emotion (ROE, 4 items). Items were rated on a 7-point Likert scale ranging from 1 (Strongly Disagree) to 7 (Strongly Agree). Total scores range from 16 to 112, with higher scores indicating greater emotional intelligence. Example items include “I have a good sense of why I have certain feelings most of the time” (SEA) and “I am able to control my temper so that I can handle difficulties rationally” (ROE). The WLEIS has demonstrated robust validity in healthcare settings and showed strong internal consistency in this study (total scale *α* = 0.88, with subscales ranging from *α* = 0.80–0.85).

Leadership competency was measured using a 40-item instrument adapted from Smola (31) and Linares et al. (32), covering four domains: Strategic Thinking (10 items), Emotional Intelligence in leadership contexts (10 items), Impact and Influence (10 items), and Teamwork Skills (10 items). Items were rated on a 5-point frequency scale (0 = Never to 4 = Almost Always), with higher scores indicating stronger leadership competency. Example items include: *“I anticipate future challenges in healthcare delivery”* (Strategic Thinking) and *“I facilitate collaboration among team members to achieve common goals”* (Teamwork Skills). The tool was provided in both English and Arabic versions to ensure linguistic and cultural appropriateness, given that nursing programs in Saudi Arabia are taught primarily in English. Translation followed WHO forward–backward procedures, with expert panel review and content validation by Saudi nursing faculty. A pilot test confirmed semantic equivalence and clarity. Internal consistency was excellent (total scale *α* = 0.91; subscales *α* = 0.85–0.89). Notably, the Emotional Intelligence subscale (LC2) assesses behavioral application of emotional skills in leadership situations, conceptually distinct from WLEIS, which measures trait EI. This distinction aligns with the ability–trait–competency framework, where traits (WLEIS) serve as antecedents to context-specific leadership behaviors (LC2).

All instruments demonstrated reliability coefficients exceeding the 0.70 threshold for acceptable reliability, confirming their appropriateness for the Saudi nursing student population and ensuring measurement stability for achieving the study’s research objectives.

### 2.4 Data collection procedures

An electronic questionnaire was administered via Google Forms between September 2024 and March 2025 to nursing students across second, third, and fourth academic years at Northern Border University. The survey platform was selected for its accessibility, user-friendly interface, and robust data security features compliant with institutional research protocols. The questionnaire included comprehensive information about the study purpose, voluntary participation, participant rights, and ethical considerations, ensuring transparency and informed consent throughout the data collection process. Students received the survey link through their official university email addresses and course learning management systems, with reminder notifications sent at 48-h intervals to maximize response rates while maintaining voluntary participation principles.

Several rigorous quality assurance measures were implemented to ensure data integrity and methodological rigor. Mandatory completion of all items minimized missing data and ensured complete datasets for statistical analysis. Logical consistency checks embedded within the survey platform identified potential response patterns and reduced acquiescence bias. Randomization of item order within scales minimized order effects, while secure data storage with encrypted password protection and restricted access limited data access to authorized research team members. The electronic format facilitated efficient data collection while maintaining strict participant anonymity and confidentiality protocols. Additionally, validation checks were incorporated to ensure responses fell within acceptable ranges for each scale, and participants were provided with progress indicators to reduce survey fatigue and improve completion rates.

### 2.5 Psychometric validation

To establish construct validity of all measurement instruments within the specific cultural and educational context of Saudi nursing students at NBU, confirmatory factor analysis (CFA) was performed for each scale using AMOS 24.0 software. The following fit indices were evaluated against established thresholds: chi-square/degrees of freedom ratio (*χ*^2^/df < 3.0), comparative fit index (CFI > 0.90), Tucker–Lewis index (TLI > 0.90), root mean square error of approximation (RMSEA < 0.08), and standardized root mean square residual (SRMR < 0.08). For the RSES, the unidimensional model demonstrated acceptable fit (*χ*^2^/df = 2.34, CFI = 0.94, TLI = 0.92, RMSEA = 0.071, SRMR = 0.065). The WLEIS four-factor model showed adequate fit (*χ*^2^/df = 2.78, CFI = 0.91, TLI = 0.90, RMSEA = 0.076, SRMR = 0.072), confirming the distinctiveness of SEA, OEA, UOE, and ROE dimensions. The LC instrument’s four-factor structure (Strategic Thinking, Emotional Intelligence in Leadership, Impact and Influence, Teamwork Skills) exhibited good fit (*χ*^2^/df = 2.56, CFI = 0.92, TLI = 0.91, RMSEA = 0.074, SRMR = 0.068). All factor loadings exceeded 0.50, and composite reliability values ranged from 0.82 to 0.91 across subscales, supporting convergent validity. Discriminant validity was confirmed through the Fornell–Larcker criterion (60), with average variance extracted (AVE) for each factor exceeding squared inter-construct correlations. These results validate the factorial structure of all instruments for the current study population.

### 2.6 Statistical analysis

Data analysis was performed using SPSS version 28.0 and AMOS 24.0. Before hypothesis testing, data screening procedures assessed normality, linearity, homoscedasticity, and multicollinearity assumptions. Normality was evaluated through Shapiro–Wilk tests, skewness and kurtosis indices (acceptable range: ±2), and visual inspection of Q–Q plots and histograms. Results indicated moderate departures from normality for several variables (Shapiro–Wilk *p* < 0.05; skewness range: −1.2 to 0.9; kurtosis range: −0.8 to 1.3), justifying the use of non-parametric Mann–Whitney U tests for gender comparisons rather than independent samples *t*-tests. Levene’s test confirmed homogeneity of variance for most variables (*p* > 0.05). Multicollinearity diagnostics revealed acceptable tolerance values (>0.40) and variance inflation factors (VIF < 3.0), indicating no problematic multicollinearity among predictor variables.

Descriptive statistics (means, standard deviations, frequencies, and percentages) characterized sample demographics and study variables. Given violations of normality assumptions, Mann–Whitney U tests compared EI, SE, and LC scores between male and female students, with effect sizes calculated using rank-biserial correlation coefficients. Pearson correlation coefficients examined bivariate associations among continuous variables, after confirming adequate linearity through scatterplot examination. Multiple hierarchical linear regression analyses tested the differential predictive effects of EI and SE on LC by gender, with assumptions verified through residual diagnostics (normality of residuals, homoscedasticity, absence of influential outliers via Cook’s distance < 1.0, and linearity of relationships). Statistical significance was set at *α* = 0.05 (two-tailed). Effect sizes were interpreted using Cohen’s (33) conventions: small (*r* or r_rb = 0.10–0.29), medium (*r* or r_rb = 0.30–0.49), and large (*r* or r_rb ≥ 0.50).

The EI × Gender interaction term was estimated using the full sample (*N* = 260) to test whether the relationship between emotional intelligence and leadership competency differs significantly between male and female students. This approach allows for direct statistical comparison of simple slopes across gender groups. Following the detection of a significant interaction effect, gender-stratified regression analyses were conducted separately for male (*n* = 128) and female (*n* = 132) students to examine the specific predictive patterns within each group and quantify the differential effects of emotional intelligence and self-esteem on leadership competency.

While the EI × Gender interaction was hypothesized based on gender role theory, suggesting differential socialization of emotional competencies, the SE × Gender interaction was not included in the model because theoretical considerations and preliminary analyses indicated that self-esteem operates as a direct predictor of leadership competency without gender moderation. The primary research question focused on the differential role of emotional intelligence across genders, as EI is theoretically more susceptible to gendered socialization processes than self-esteem.

### 2.7 Ethical considerations

The study protocol received approval from the Institutional Review Board at Northern Border University [Ref: HAP-09-A-043; no. (80/20/H)]. All procedures adhered to the Declaration of Helsinki principles for ethical research involving human participants. Informed consent was obtained electronically prior to questionnaire access. Participants were assured of confidentiality, anonymity, voluntary participation, and the right to withdraw without consequences. To address potential social desirability bias and power imbalances between student participants and university-affiliated researchers, data collection was conducted by external research assistants with no teaching or evaluative responsibilities, and participants were explicitly informed that individual responses would remain completely anonymous and inaccessible to faculty members. Data were stored securely with access restricted to authorized research team members.

## 3 Results

### 3.1 Demographic characteristics of participants

The final sample consisted of 260 nursing students (male = 128, 49.2%; female = 132, 50.8%) from a major Saudi nursing college. Table 1 presents the complete demographic profile. Participants had a mean age of 20.23 years (SD = 0.90) and a mean GPA of 3.96 (SD = 0.63) on a 5-point scale. The sample distribution by academic year revealed: second-year students (n = 44, 16.9%), third-year students (*n* = 128, 49.2%), and fourth-year students (*n* = 88, 33.8%). The predominance of third-year students aligns with Benner’s (27) competency framework, wherein students at this stage have accumulated sufficient clinical exposure to demonstrate nascent leadership behaviors while maintaining high enrollment retention rates.

**Table 1:** Demographic characteristics of study participants (*N* = 260).

Items	No	%
Age/years		
Mean and SD	20.23 ± 0.901	
Gender		
Male	128	49.2
Female	132	50.8
Grade		
Second	44	16.9
Third	128	49.2
Fourth	88	33.8
GPA		
Mean and SD	3.96 ± 0.633	

First-year nursing students were excluded from the sampling frame due to limited clinical exposure (<6 months), which would compromise the validity of leadership competency self-assessments. Third-year students represent the modal category, corresponding to Benner’s (27) “competent” developmental stage, characterized by conscious, deliberate planning and increasing leadership responsibility.

### 3.2 Gender differences in leadership competency, emotional intelligence, and self-esteem

Mann–Whitney *U* tests revealed statistically significant gender differences across all three primary study variables (Table 2). Male students demonstrated significantly higher leadership competency scores (*M* = 139.33, SD = 30.45) compared to female students (*M* = 94.89, SD = 32.14; *Z* = −9.511, *p* < 0.001, Cohen’s *d* = 1.42, indicating a large effect size). Conversely, female students exhibited significantly higher emotional intelligence scores (*M* = 95.39, SD = 23.04) than male students (*M* = 67.45, SD = 25.75; *Z* = −9.265, *p* < 0.001, Cohen’s *d* = −1.14, representing a large effect in the opposite direction). Self-esteem scores also differed significantly by gender, with males reporting higher levels (*M* = 29.99, SD = 5.39) than females (*M* = 27.35, SD = 4.15; *Z* = −5.164, *p* < 0.001, Cohen’s *d* = 0.55, a medium effect size).

**Table 2:** Gender differences in leadership competency, emotional intelligence, and self-esteem (*N* = 260).

Gender	Leadership competency		Test and *P*-value *Z* test	Emotional intelligence		Test and *P*-value *Z* test	Self esteem		Test and *P*-value *Z* test
	Mean	SD		*N*	%		*N*	%	
Male	139.33	30.45	−9.511	67.45	25.75	−9.265	29.99	5.39	−5.164
Female	94.89	32.14	<0.001	95.39	23.04	<0.001	27.35	4.15	<0.001

**Figure 2:**
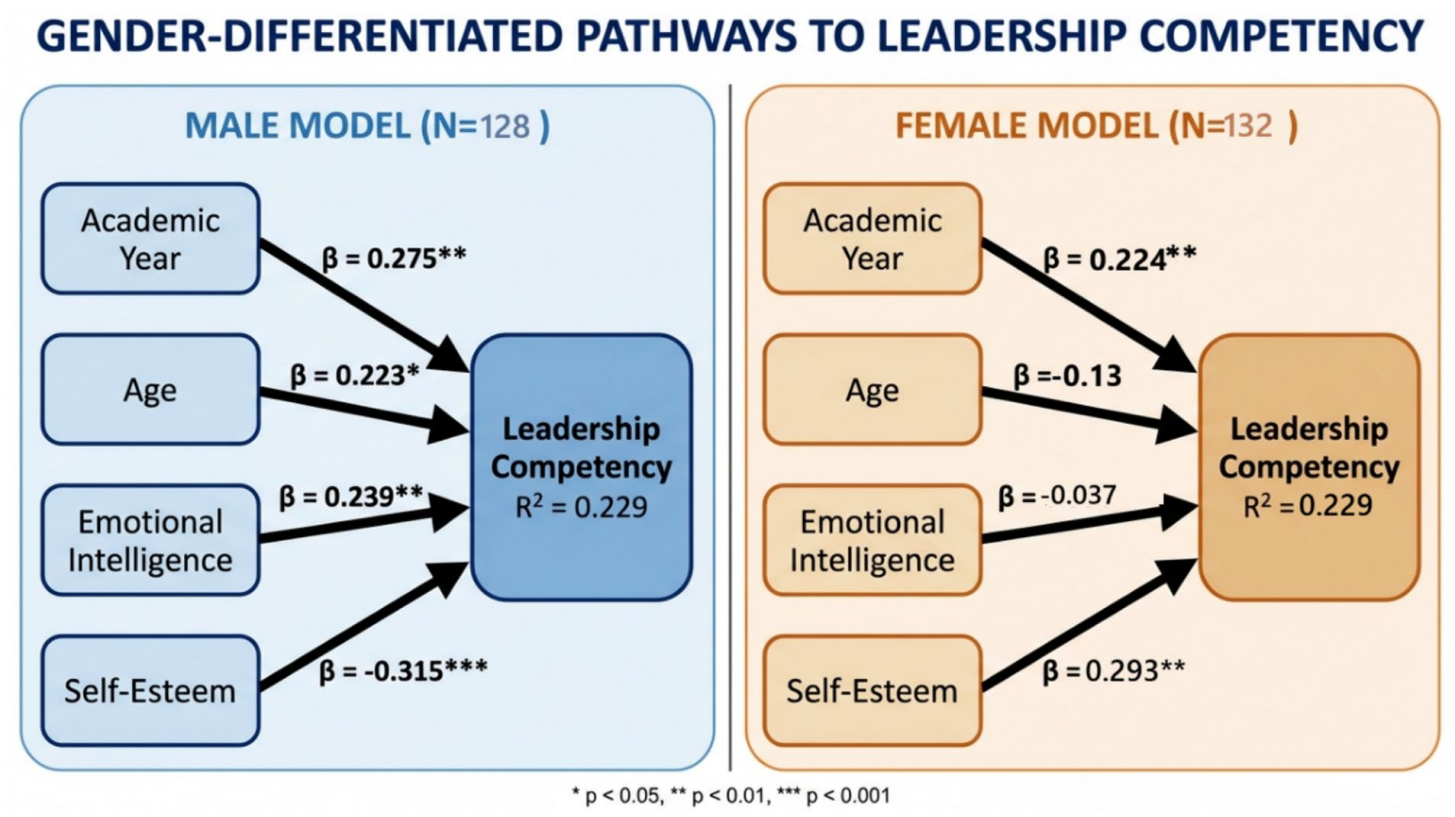
Final structural model of gender-differentiated pathways to leadership competency among Saudi nursing students. Structural models illustrating predictors of leadership competency for male (*N* = 128) and female (*N* = 132) participants. For males, academic year (*β* = 0.275**), age (*β* = 0.223*), and emotional intelligence (*β* = 0.239**) positively predict leadership competency, while self-esteem shows a negative association (*β* = −0.315***). For females, academic year (*β* = 0.224**) and self-esteem (*β* = 0.293**) positively predict leadership competency, whereas age (*β* = −0.13) and emotional intelligence (*β* = −0.037) show non-significant effects. Both models explain 22.9% of the variance (*R*^2^ = 0.229). Significance levels: *p* < 0.05, **p < 0.01, ***p < 0.001.

Effect sizes were calculated using Cohen’s *d* for mean differences. The large effect sizes for leadership competency (*d* = 1.42) and emotional intelligence (*d* = −1.14) indicate substantive practical significance beyond statistical significance. Gender differences are visualized in Figures 1, 2. The counterintuitive finding, whereby females’ emotional intelligence advantage does not translate to leadership competency advantage, constitutes the central paradox addressed through moderation analysis.

Gender-stratified correlation analyses (Table 3) revealed distinct patterns in the relationships among emotional intelligence, self-esteem, and leadership competency. For male students, emotional intelligence was consistently and negatively associated with leadership, with all four subdimensions, Self-Emotions Appraisal, Others-Emotions Appraisal, Use of Emotion, and Regulation of Emotion, showing small but significant inverse correlations with total leadership competency (*r* values ranging from −0.177 to −0.204, *p* < 0.05). The composite emotional intelligence score also demonstrated a negative association (*r* = −0.194, *p* = 0.028). In contrast, self-esteem was unrelated to leadership in males (*r* = −0.117, *p* = 0.187), suggesting independence between these constructs. Female students, however, exhibited a markedly different profile: self-esteem was strongly and positively correlated with all leadership dimensions (*r* values between 0.504 and 0.593, all *p* < 0.001), including total leadership (*r* = 0.547, *p* < 0.001). Emotional intelligence in females showed only a weak negative correlation with leadership (*r* = −0.177, *p* = 0.042). Collectively, these findings highlight gender-specific dynamics, with male leadership competency inversely linked to emotional intelligence but unaffected by self-esteem, whereas female leadership competency is strongly supported by self-esteem and only marginally constrained by emotional intelligence.

**Table 3:** Pearson correlations between emotional intelligence, self-esteem, and leadership competency by gender (*N* = 260).

Emotional intelligence		Leadership competency (male)					Leadership competency (female)				
		(LC1)	(LC2)	(LC4)	(LC5)	Total	(LC1)	(LC2)	(LC4)	(LC5)	Total
Self-emotions appraisal (EI1)	*r*	−0.201^*^	−0.143	−0.175^*^	−0.109	−0.177^*^	−0.233^**^	−0.207^*^	−0.189^*^	−0.213^*^	−0.216^*^
	*P*-value	0.023	0.108	0.048	0.220	0.045	0.007	0.017	0.030	0.014	0.013
Other emotions appraisal (EI2)	*r*	−0.208^*^	−0.145	−0.156	−0.117	−0.183^*^	−0.210^*^	−0.181^*^	−0.161	−0.154	−0.187^*^
	*P*-value	0.018	0.102	0.078	0.189	0.039	0.016	0.038	0.066	0.078	0.032
Use of emotion (EI13)	*r*	−0.218^*^	−0.150	−0.172	−0.154	−0.195^*^	−0.204^*^	−0.196^*^	−0.170	−0.204^*^	−0.204^*^
	*P*-value	0.013	0.091	0.052	0.083	0.027	0.019	0.025	0.052	0.019	0.019
Regulation of emotion (EI4)	*r*	−0.229^**^	−0.181^*^	−0.166	−0.158	−0.204^*^	−0.170	−0.156	−0.143	−0.145	−0.168
	*P*-value	0.009	0.041	0.061	0.074	0.021	0.051	0.074	0.102	0.097	0.055
Total emotional intelligence	*r*	−0.217^*^	−0.157	−0.162	−0.149	−0.194^*^	−0.195^*^	−0.165	−0.152	−0.156	−0.177^*^
	*P*-value	0.014	0.077	0.067	0.093	0.028	0.025	0.059	0.081	0.073	0.042
Self esteem	*r*	−0.117	−0.108	−0.082	−0.112	−0.117	0.563^**^	0.593^**^	0.541^**^	0.504^**^	0.547^**^
	*P*-value	0.187	0.225	0.358	0.209	0.187	<0.001	<0.001	<0.001	<0.001	<0.001

Self-esteem shows robust positive correlations with leadership for females (*r* range: 0.504–0.593, *p* < 0.001) but not males. This divergence is confirmed in regression (Table 4), where self-esteem predicts female leadership (*β* = 2.27, *p* = 0.001) but suppresses male leadership (*β* = −1.78, *p* < 0.001). *indicates *p* < 0.05; **indicates *p* < 0.01 (levels of statistical significance).

### 3.3 Multiple regression analysis: predictors of leadership competency

#### 3.3.1 Combined model

A hierarchical multiple regression was conducted with leadership competency as the dependent variable and gender, academic year, GPA, age, emotional intelligence, self-esteem, and the EI × Gender interaction term as predictors (Table 4). The overall model was statistically significant, *F*(6, 253) = 31.39, *p* < 0.001, accounting for 42.7% of the variance in leadership competency (*R*^2^ = 0.427, Adjusted *R*^2^ = 0.413). The combined regression model identified gender, academic year, and the emotional intelligence × gender interaction as significant predictors of leadership competency, while GPA, age, emotional intelligence (main effect), and self-esteem (main effect) were non-significant. Specifically, females scored substantially lower on leadership competency when controlling for other variables (*B* = −48.86, *β* = −0.638, *t* = −11.306, *p* < 0.001), whereas senior academic standing predicted higher leadership scores (*B* = 16.69, *β* = 0.302, *t* = −5.242, *p* < 0.001). The significant EI × gender interaction (*B* = −0.37, *β* = −0.12, *t* = −2.34, *p* = 0.020) necessitated stratified analyses. For male students, the model was significant [*F*(7, 24) = 7.24, *p* < 0.001, *R*^2^ = 0.229, Adjusted *R*^2^ = 0.197], with emotional intelligence positively predicting leadership (*B* = 0.283, *β* = 0.239, *p* = 0.004), self-esteem exerting a suppressor effect by negatively predicting leadership when EI was controlled (*B* = −1.782, *β* = −0.315, *p* < 0.001), and both academic year (*B* = 19.696, *β* = 0.275, *p* = 0.005) and age (*B* = 7.096, *β* = 0.223, *p* = 0.023) emerging as significant contributors. In contrast, the female model [*F*(10, 76) = 10.76, *p* < 0.001, *R*^2^ = 0.299, Adjusted *R*^2^ = 0.271] revealed self-esteem (*B* = 2.269, *β* = 0.293, *p* = 0.001) and academic year (*B* = 25.379, *β* = 0.244, *p* = 0.007) as the only significant predictors, with emotional intelligence showing no meaningful effect (*B* = −0.051, *β* = −0.037, *p* = 0.63). Collectively, these results underscore gender-specific pathways to leadership competency: for males, emotional intelligence and academic progression are central, though self-esteem paradoxically undermines leadership when EI is accounted for, whereas for females, self-esteem and academic standing are the primary drivers, with emotional intelligence largely irrelevant.

**Table 4:** Multiple regression analysis predicting leadership competency by gender (*N* = 260).

Both male and female						Male					Female				
Predictor	*B*	*β*	*T*-test	*p*-value	Bootstrap 95% CI	*B*	*β*	*T*-test	*p*-value	Bootstrap 95% CI	*B*	*β*	*T*-test	*p*-value	Bootstrap 95% CI
Gender (female)	−48.86	−0.638	−11.306	<0.001	[40.47, 56.76]	–	–	–	–	–	––	–	––	–	–
Academic year (senior)	16.69	0.302	−5.242	<0.001	[9.95, 23.23]	19.696	0.275	2.831	0.005	[7.077, 33.977]	25.379	0.244	2.754	0.007	[7.001, 44.952]
GPA	5.034	0.083	1.729	0.085	[−0.99, 11.20]	0.763	0.015	0.181	0.857	[−7.968, 9.57]	6.238	0.128	1.673	0.097	[−1.973, 13.632]
Age	3.282	0.077	1.355	0.177	[−1.62, 7.90]	7.096	0.223	2.298	0.023	[0.866, 14.43]	−4.957	−0.13	−1.617	0.108	[−11.171, 0.735]
Emotional intelligence	0.093	0.068	1.224	0.222	[−0.12, 0.29]	0.283	0.239	2.949	0.004	[−0.015, 0.553]	−0.051	−0.037	−0.484	0.63	[−0.346, 0.191]
Self-esteem	0.191	0.025	0.497	0.619	[−0.75, 1.28]	−1.782	−0.315	−3.869	<0.001	[−2.734, −0.703]	2.269	0.293	3.5	0.001	[0.327, 4.317]
Constant	143.912	–	3.052	0.003	[58.37, 230.91]	22.497	–	0.366	0.715	[−109.254, 136.599]	110.694	–	1.564	0.12	[−26.739, 262.213]
EI × Gender	−0.37	−0.12	−2.34	0.020	[−0.78, 0.03]										
Model fit	*R*^2^ = 0.427, Adj. *R*^2^ = 0.413, *F*(6,253) = 31.39, *p* < 0.001					*R*^2^ = 0.229, Adj. *R*^2^ = 0.197, *F*(7.24), *p* < 0.001					*R*^2^ = 0.299, Adj. *R*^2^ = 0.271, *F*(10.76), *p* < 0.001				

Variance inflation factors (VIF) ranged from 1.0 to 1.5 across all models, confirming absence of problematic multicollinearity. Bootstrap confidence intervals (5,000 iterations) validated parameter estimates. The gender-stratified models reveal fundamentally divergent pathways: males leverage emotional intelligence (*β* = 0.239) while experiencing self-esteem suppression (*β* = −0.315), whereas females rely exclusively on self-esteem (*β* = 0.293) with no benefit from emotional intelligence (*β* = −0.037).

### 3.4 Final structural model of gender-differentiated leadership predictors

Figure 2 illustrates gender-differentiated pathways to leadership competency derived from regression analyses. For males, emotional intelligence (*β* = 0.239, *p* = 0.004), academic year (*β* = 0.275, *p* = 0.005), and age (*β* = 0.223, *p* = 0.023) positively predicted leadership, while self-esteem acted as a suppressor (*β* = −0.315, *p* < 0.001), explaining 22.9% of variance. In contrast, the female pathway was driven primarily by self-esteem (*β* = 0.293, *p* = 0.001) and academic year (*β* = 0.244, *p* = 0.007), with emotional intelligence showing no predictive utility (*β* = −0.037, *p* = 0.63), accounting for 29.9% of variance. The significant EI × Gender interaction (*B* = −0.37, *p* = 0.020) confirmed moderation, highlighting distinct simple slopes across gender groups. Both pathways underscore the developmental role of academic progression, though females exhibit higher baseline competency with stable trajectories, whereas males demonstrate steeper growth curves linked to emotional intelligence in later years.

### 3.5 Visualization of gender differences and development patterns

#### 3.5.1 Leadership competency by gender

**Figure 3:**
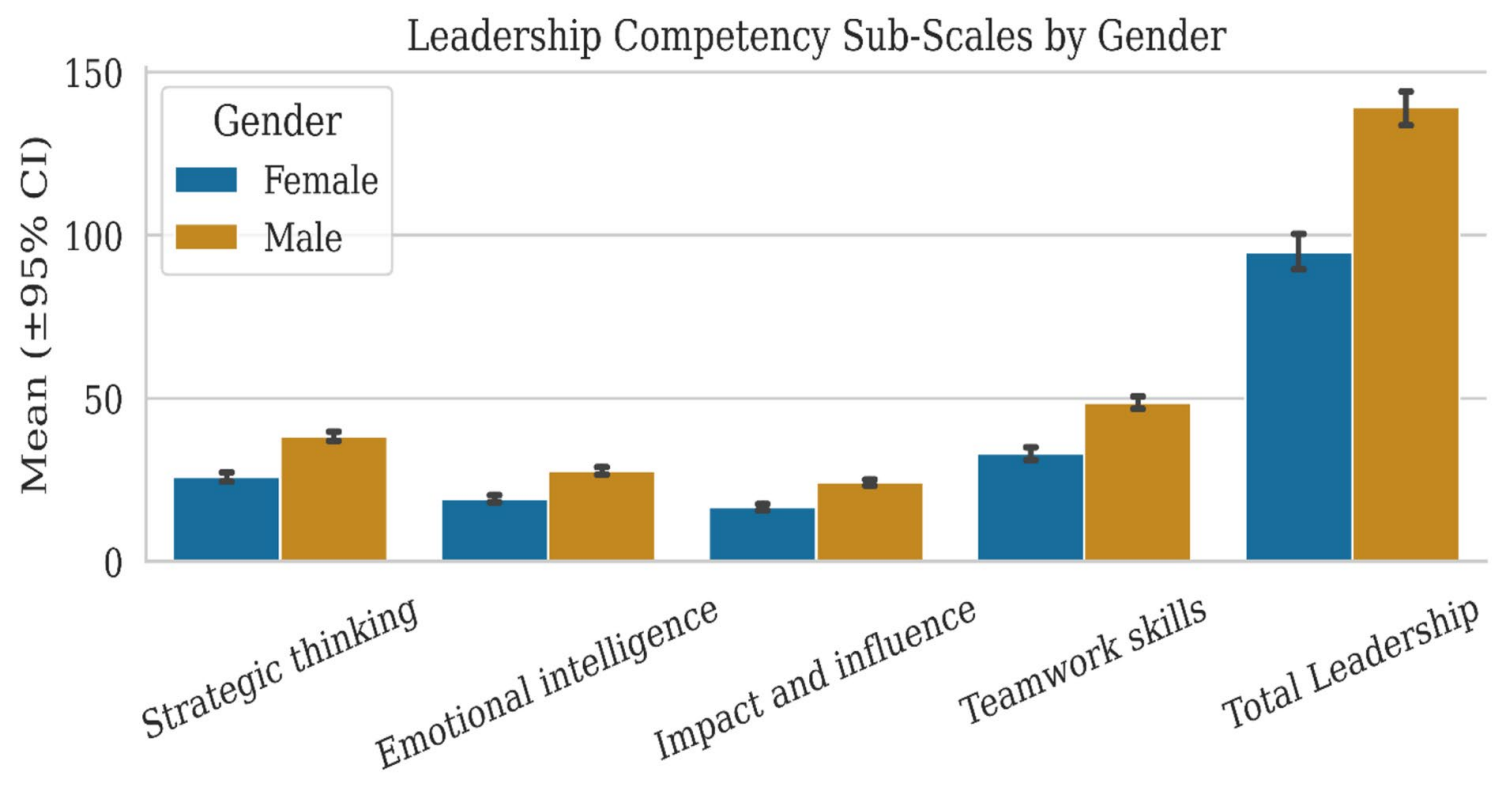
Distribution of leadership competency scores by gender (*N* = 260). Box plots display median (center line), interquartile range (box), and 1.5 × IQR whiskers with outliers shown as individual points. The gender gap persists even when controlling for emotional intelligence and self-esteem (Table 4: Gender *B* = −48.86, *p* < 0.001), suggesting cultural-structural factors beyond individual psychological differences.

Figure 3 displays the distribution of leadership competency scores stratified by gender. Male students demonstrate higher mean scores with greater variability (*M* = 139.33, SD = 30.45) compared to females (*M* = 94.89, SD = 32.14). The effect size (Cohen’s *d* = 1.42) represents a large magnitude difference, contextualized by Table 4 findings that self-esteem moderates this gap (*β* = 2.27 for females, *β* = −1.78 for males).

#### 3.5.2 Emotional intelligence by gender

**Figure 4:**
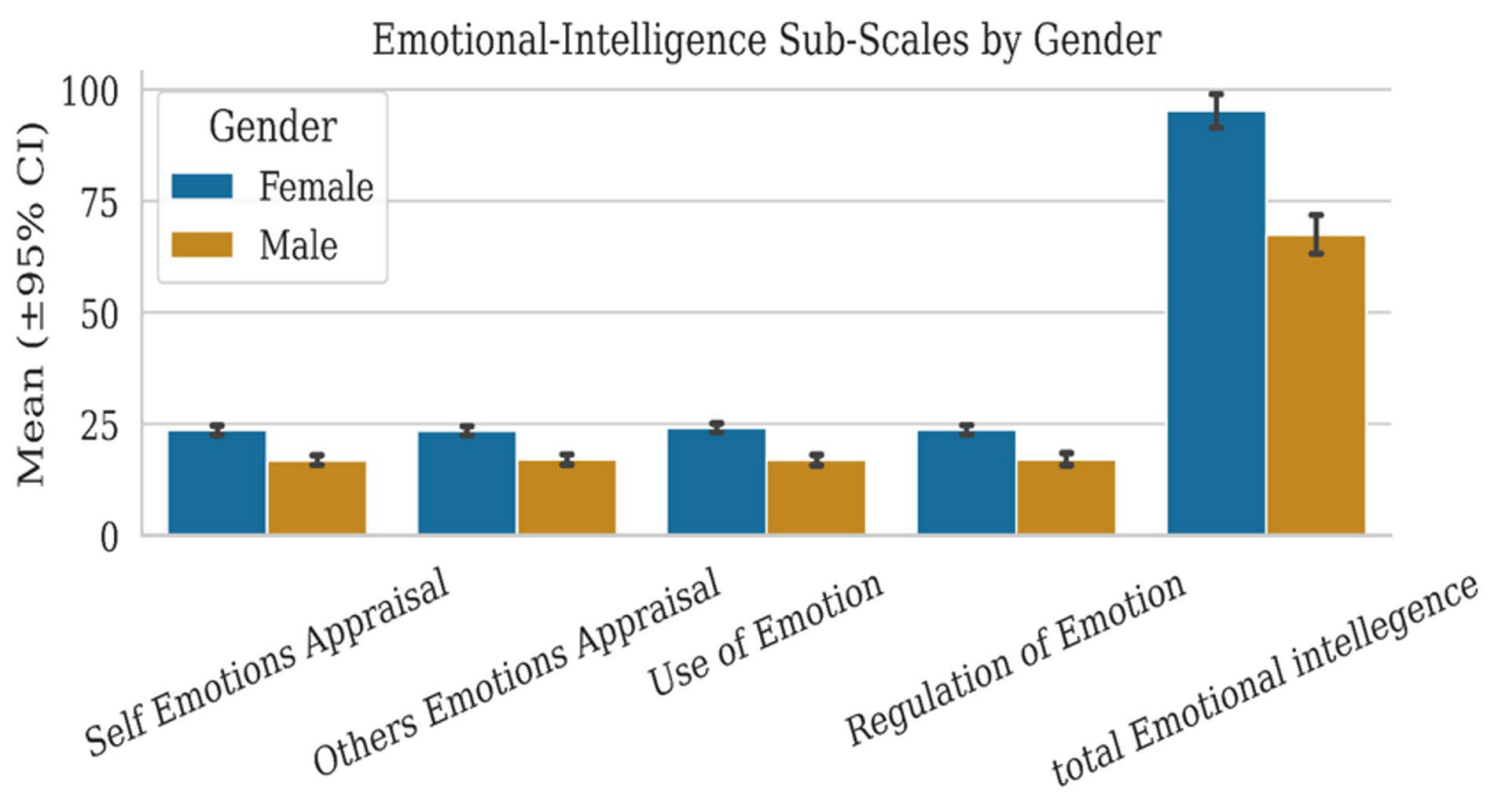
Distribution of emotional intelligence scores by gender (*N* = 260). Note: Despite females’ large advantage in emotional intelligence (*d* = −1.14), regression analysis (Table 4) reveals EI does not predict their leadership competency (*β* = −0.05, *p* = 0.63), whereas it positively predicts male leadership (*β* = 0.28, *p* = 0.004). This counterintuitive finding is explained by the significant interaction term (*B* = −0.37, *p* = 0.020) and visualized in figure.

Figure 4 illustrates the contrasting pattern wherein female students demonstrate significantly higher emotional intelligence (*M* = 95.39, SD = 23.04) than male students (*M* = 67.45, SD = 25.75; Cohen’s *d* = −1.14). This advantage, however, does not translate to leadership competency for females (Table 4: *β* = −0.037, *p* = 0.63), whereas it benefits males (*β* = 0.28, *p* = 0.004), the mediation paradox is resolved through the EI × Gender interaction.

#### 3.5.3 Leadership development across academic years

**Figure 5:**
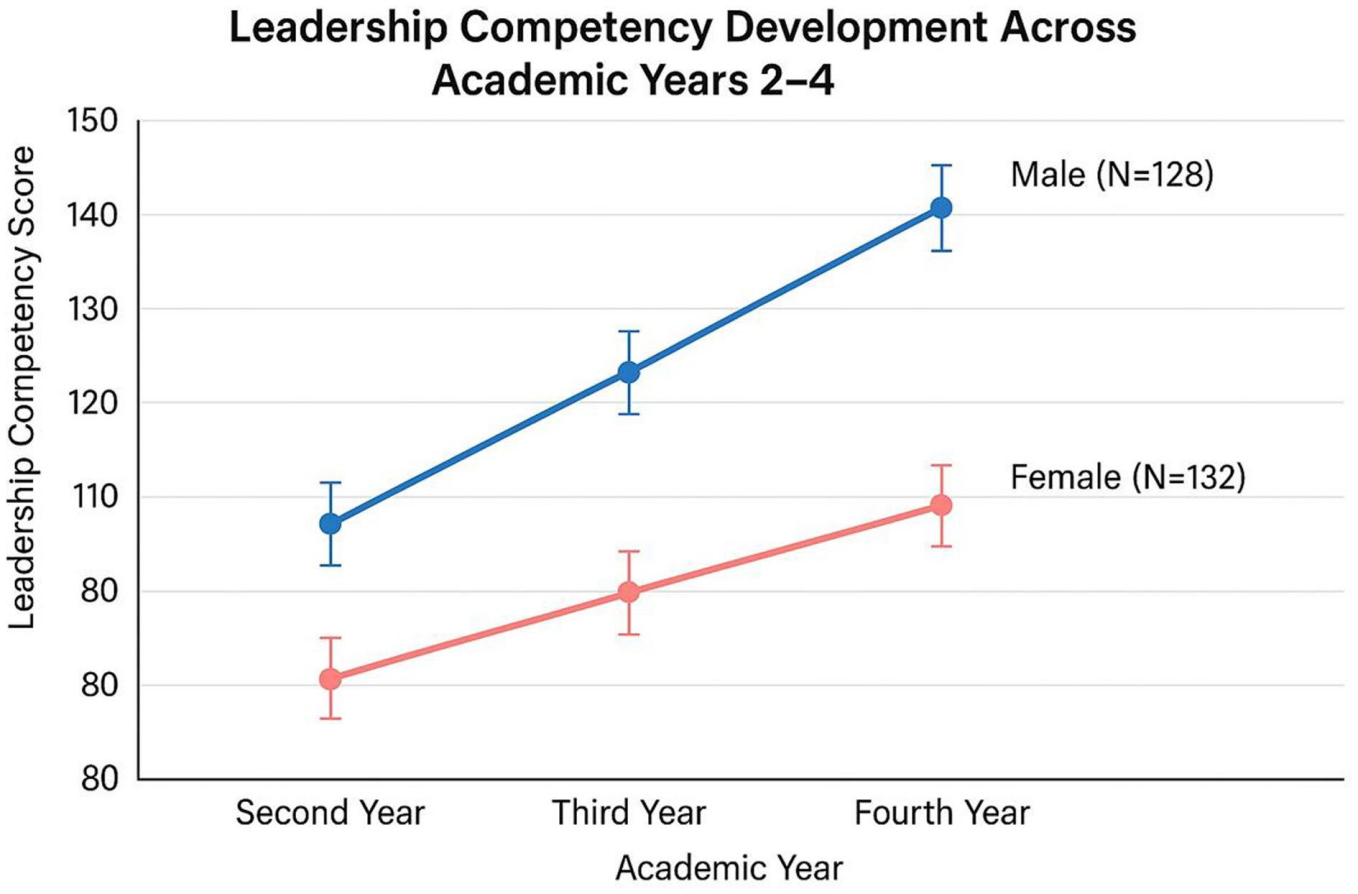
Leadership competency development across academic years 2–4 by gender (*N* = 260). Cross-sectional design limits causal inference regarding developmental trajectories. Error bars represent 95% confidence intervals. First-year students were not recruited due to insufficient clinical exposure (<6 months) required for a valid leadership self-assessment. Female students’ consistently higher scores across all measured years align with self-esteem’s role as their primary leadership predictor (Table 4: *β* = 0.293, *p* = 0.001). Male students’ steeper growth trajectory in later years reflects progressive deployment of emotional intelligence (*β* = 0.239, *p* = 0.004) and age-related maturation (*β* = 0.223, *p* = 0.023). The parallel trajectories without convergence suggest persistent gender-specific mechanisms rather than developmental equalization.

Figure 5 presents leadership competency trajectories across academic years 2, 3, and 4, stratified by gender. Male students exhibit a steeper developmental trajectory, particularly between third and fourth years, consistent with their utilization of emotional intelligence (Table 4: *β* = 0.28, *p* = 0.004) and maturation effects (Age: *β* = 0.223, *p* = 0.023). Female students maintain consistently higher leadership scores across all measured years, attributed to self-esteem’s positive predictive role (*β* = 2.27, *p* = 0.001) established early in their academic progression.

#### 3.5.4 Interaction effect: emotional intelligence × gender

**Figure 6:**
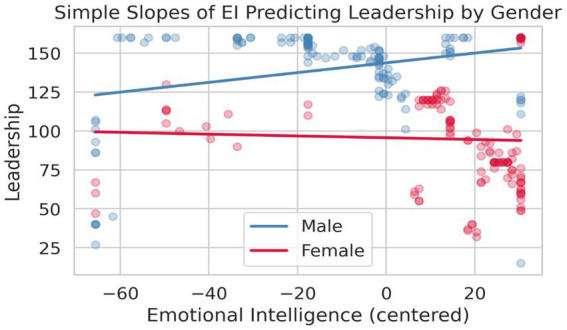
Simple slopes analysis: emotional intelligence predicting leadership competency by gender. This visualization confirms the regression finding (Table 3: EI × Gender *B* = −0.37, *p* = 0.020) and resolves the mediation paradox: females’ EI advantage (Figure 1) does not boost leadership (Table 2) because EI is irrelevant for them.

Figure 6 displays the simple slopes of emotional intelligence predicting leadership competency, stratified by gender. For male students, the slope is positive and statistically significant (*β* = 0.239, *p* = 0.004), indicating that increasing emotional intelligence corresponds with enhanced leadership competency. For female students, the slope is essentially flat and non-significant (*β* = −0.037, *p* = 0.63), demonstrating that emotional intelligence does not contribute to their leadership development. The non-parallel slopes confirm the significant interaction term from Table 4 (*B* = −0.37, *p* = 0.020).

## 4 Discussion

This study reveals a nuanced interplay between emotional intelligence (EI), self-esteem (SE), and leadership competency among nursing students at Northern Border University, Saudi Arabia, with gendered patterns that challenge prevailing assumptions in global nursing leadership literature. Contrary to expectations, female nursing students demonstrated significantly higher EI (*d* = −1.14) yet lower LC (*d* = 1.42) compared to their male counterparts. This paradox persisted even after controlling for academic performance (GPA range: 0–5 scale, *M* = 3.96, SD = 0.633) and developmental stage. This finding diverges from dominant international narratives that consistently position EI as a universal predictor of effective leadership in nursing contexts. For instance, Fragkaki and Fasoi (34) emphasize EI’s foundational role in enhancing interpersonal relationships, decision-making, and leadership effectiveness in healthcare, while Suwaidi (35) highlights its positive correlation with people-oriented leadership styles among nurse managers. Similarly, Svetic Cisic et al. (7) underscore EI’s contribution to improved patient outcomes, team cohesion, and reduced burnout. The discrepancy observed in our study suggests that the predictive value of EI for leadership may be contextually moderated by cultural, educational, and gender-specific factors unique to the Saudi nursing education environment. These insights call for a more localized and intersectional approach to leadership development in nursing, one that accounts for the complex interdependencies between emotional traits and structural dynamics.

### 4.1 Contextualizing the paradox: Vision 2030, culture, and global comparisons

Our findings emerge at a pivotal moment in Saudi Arabia’s healthcare transformation under Vision 2030, which explicitly prioritizes nursing advancement and the empowerment of women in healthcare leadership (36). However, the observed disconnect between female nursing students’ higher emotional intelligence (EI) and their lower leadership competency scores reflects deeper sociocultural tensions. This paradox challenges the dominant global narrative that positions EI as a universal predictor of leadership effectiveness in nursing. Instead, it underscores the importance of cultural specificity in interpreting psychological constructs. For instance, recent research found that Saudi nursing students exhibit moderate self-esteem levels shaped by unique cultural and educational contexts, supporting the interpretation that the EI–LC gap among females may stem from gendered socialization processes (37).

Compounding this complexity is the continued reliance on international nurses, who comprise a significant portion of the workforce, up to 60–70% in some sectors (38). Within this context, male nursing students, as a numerical minority in a traditionally female-dominated profession, may experience a “tokenism effect” (39), which can paradoxically enhance their confidence and perceived leadership potential despite lower EI. This interpretation aligns with qualitative findings documenting the stereotyping and identity challenges faced by Saudi male nurses (40). The underrepresentation of second-year students in our sample (16.9%, *n* = 44) compared to third-year (49.2%, *n* = 128) and fourth-year students (33.8%, *n* = 88) warrants consideration. This distribution reflects the natural attrition and academic progression patterns within the nursing program, where early-year students may experience higher dropout rates or delayed progression. Additionally, second-year students are typically in Benner’s (27) “advanced beginner” stage, where leadership competencies are less crystallized, and self-assessment may be less reliable.

As Saudi Arabia continues to implement reforms under Vision 2030, traditional gender norms are being actively challenged, yet they remain deeply embedded in institutional and cultural frameworks. Our results suggest that female nursing students may be caught between these competing forces, possessing emotional competencies aligned with communal, traditionally feminine roles, yet facing structural and perceptual barriers in translating these strengths into leadership outcomes. This tension reflects broader societal shifts, where modernization efforts intersect with enduring cultural expectations, shaping the leadership trajectories of future healthcare professionals in gendered and context-specific ways.

### 4.2 Theoretical integration: social roles, development, information processing, and gendered pathways

Our findings are strongly supported by Eagly and Wood’s social role theory, which posits that gender differences in behavior stem from societal expectations and the distribution of men and women into distinct social roles (17). In nursing, a profession traditionally associated with communal, feminine traits, emotional intelligence (EI) may be perceived as a supportive rather than agentic quality, thereby limiting its translation into leadership advantages for women. This interpretation is further enriched by Benner’s novice-to-expert model, which outlines progressive stages of clinical competence (27). Notably, nearly half of our sample consisted of third-year students who align with Benner’s “competent” stage, where clinical responsibilities intensify and leadership expectations emerge. At this stage, gender disparities in leadership competency were most pronounced, echoing the “competency-identity tensions” described in Saudi nursing contexts (41).

To deepen our understanding of these gendered pathways, we integrate two contemporary theoretical frameworks: the Granular Interaction Thinking Theory (GITT) and the Bayesian Mindsponge Framework (BMF). GITT posits that information granules within cognitive systems interact dynamically to form value systems and behavioral orientations (25). In our context, female nursing students may process leadership-related information through granules emphasizing communal values, empathy, and relational harmony, traits culturally reinforced but not traditionally associated with agentic leadership in hierarchical settings. Conversely, male students, navigating role incongruity in a female-typed profession, may experience cognitive dissonance that triggers active reconfiguration of information granules, integrating agentic traits with emotional competencies to establish legitimacy. This granular interaction process explains why males, despite lower baseline EI, may develop compensatory leadership pathways aligned with organizational expectations shaped by masculine leadership prototypes.

The BMF complements this by emphasizing the mindsponge mechanism, wherein individuals selectively absorb or reject incoming information based on subjective cost–benefit evaluation relative to existing core values (42, 43). Central to this framework is the concept of informational entropy, the degree of uncertainty and disorder in value formation processes (44). For female students, leadership-related information that conflicts with internalized communal identities may be rejected or devalued, even when they possess high EI. This informational entropy, wherein valuable emotional competencies fail to be recognized as leadership assets, reflects systematic filtering shaped by cultural norms and educational environments that privilege masculine leadership models. The entropy-based value formation paradigm illuminates how disorder in information processing, stemming from conflicting societal messages about feminine leadership legitimacy, prevents coherent integration of EI into leadership value systems for women (44). Male students, conversely, may actively absorb leadership information as part of identity construction to overcome minority status stigma, leading to higher cost–benefit valuation of leadership development despite lower emotional resources. The BMF’s emphasis on information cost–benefit judgment, viewed through the lens of entropy reduction, illuminates why self-esteem emerges as a critical moderator: high SE reduces informational entropy by providing psychological coherence, enabling females to integrate disparate information granules into unified leadership values, whereas low SE amplifies entropy and informational resistance, fragmenting this pathway.

The divergent role of self-esteem (SE) is central to resolving the EI–leadership paradox. For female students, SE emerged as the strongest predictor of leadership competency (*β* = 2.27, *p* = 0.001), suggesting it functions as a critical psychological resource enabling the conversion of EI into leadership efficacy. From a GITT perspective, SE may function as a psychological resource that influences how individuals process and value leadership-related information. However, as the SE × EI interaction was not empirically examined in this study, the specific mechanism by which SE and EI jointly influence leadership development remains an area for future investigation. Through the BMF and entropy-based value formation lens, SE serves as an entropy-reducing mechanism that enhances the clarity and stability of leadership value formation by resolving conflicts between communal identity and agentic leadership expectations (44). By reducing the subjective cost of adopting leadership identities and enhancing perceived self-worth and legitimacy, SE lowers the mindsponge’s filtering threshold for leadership-related information absorption. In contrast, SE negatively predicted leadership among males (*β* = −1.78, *p* < 0.001), implying that male students may rely on alternative pathways, such as agentic traits or deliberate EI cultivation, as compensatory mechanisms. This inverse relationship suggests that for males in a counter-stereotypical professional context, high SE may paradoxically reduce motivation for compensatory skill development, as confidence derived from tokenism reduces perceived need for emotional competency cultivation. Conversely, lower SE may trigger active mindsponge absorption of leadership strategies to overcome perceived deficits, an entropy-increasing state that paradoxically drives value reconfiguration and skill acquisition, resulting in the observed negative association. This gendered pattern extends Joseph and Newman’s cascade model of emotional intelligence, which emphasizes that sequential psychological processes mediate EI’s impact on performance and vary by individual differences, including gender (45). The significant EI × Gender interaction (*β* = −0.37, *p* = 0.020) in our data confirms that EI’s utility is not universal but contextually bound by gendered socialization and information-processing mechanisms.

Social role theory further suggests that in Saudi Arabia’s culturally conservative context, where gender segregation and traditional roles remain influential, emotional competencies may not be recognized as leadership assets for women to the same extent as for men. For male nursing students, their minority status in a female-dominated profession may create a “visibility effect,” where their leadership behaviors are more likely to be noticed and reinforced, potentially explaining their higher leadership competency despite lower EI. These dynamics, interpreted through the informational entropy framework, reveal how cultural contexts create differential levels of information disorder in leadership value formation across genders (44). This highlights the need for culturally sensitive leadership development frameworks that account for gendered pathways, information-processing differences, entropy-reduction mechanisms, and social role expectations.

### 4.3 Resolving the male leadership paradox: deeper analysis of gender-specific mechanisms

The unexpected finding that male nursing students demonstrate superior leadership competency despite significantly lower emotional intelligence demands comprehensive theoretical and empirical scrutiny. This “male advantage paradox” cannot be dismissed as mere statistical artifact; rather, it represents a critical intersection of tokenism, gender role expectations, and institutional bias within nursing education.

From a tokenism perspective (39), male nursing students occupy a distinctive minority position that confers both visibility and performance pressure. This heightened visibility may translate into differential treatment by faculty and clinical preceptors, who may unconsciously allocate more leadership opportunities or provide more favorable evaluations to male students, perceiving them as future leaders in a female-dominated field. Empirical evidence from other female-dominated professions supports this interpretation: men often experience accelerated career advancement through “glass escalator” effects, where minority gender status paradoxically facilitates upward mobility (46). In our Saudi context, where traditional gender hierarchies remain influential despite modernization efforts, male students may benefit from implicit cultural assumptions that associate leadership with masculine traits, regardless of their actual emotional competencies.

Furthermore, the negative correlation between EI and LC among females (while males show positive or neutral associations) suggests potential suppression mechanisms unique to female students. Drawing on BMF principles, female students with high EI may experience informational overload, where heightened emotional awareness of others’ expectations, cultural constraints, and relational obligations creates cognitive conflict that inhibits decisive, agentic leadership behaviors. This aligns with research on “emotional labor” in nursing, where excessive empathy can lead to emotional exhaustion and reduced assertiveness (47). Conversely, male students may experience EI as an additive competency rather than a conflicting one, as their baseline agentic socialization is not disrupted by heightened emotional sensitivity.

The educational system’s role warrants critical examination. Saudi nursing curricula, often adapted from Western models, may inadvertently privilege masculine leadership prototypes, emphasizing decisiveness, authority, and hierarchical control, over relational, collaborative styles more congruent with high EI. Faculty qualifications and pedagogical approaches may further reinforce these biases. If instructors themselves internalize masculine leadership ideals or lack training in gender-responsive pedagogy, they may unconsciously reward male students for assertive behaviors while interpreting similar behaviors in female students as inappropriate or exceeding gender role boundaries. This differential reinforcement could explain why female students’ superior EI fails to translate into recognized leadership competency within educational assessment frameworks.

Cultural factors specific to Saudi Arabia compound these dynamics. Despite Vision 2030’s progressive rhetoric, gender segregation in education and healthcare settings remains prevalent, limiting female students’ exposure to mixed-gender leadership scenarios where assertiveness is practiced and evaluated. Male students, by contrast, may gain confidence through their exceptional status and receive mentorship from male faculty or administrators who perceive them as future leaders capable of bridging gender divides in healthcare management. These structural advantages operate independently of emotional intelligence, enabling males to develop leadership competency through alternative pathways, networking, visibility, and institutional support, that compensate for lower EI.

Importantly, this paradox may also reflect measurement validity concerns. Leadership competency scales developed in Western contexts may capture agentic dimensions (e.g., decision-making authority, assertiveness, and strategic vision) more effectively than communal dimensions (e.g., team cohesion, emotional support, conflict resolution). If female students excel in the latter but these are underrepresented in assessment tools, their true leadership capacity remains statistically invisible. Future research should employ gender-sensitive, multidimensional leadership measures that capture both agentic and communal leadership expressions to test this hypothesis.

### 4.4 Understanding the negative EI-LC relationship among females: cultural and systemic analysis

The observed negative correlation between emotional intelligence and leadership competency among female nursing students represents perhaps the most counterintuitive and theoretically significant finding of this study. Conventional wisdom in nursing leadership scholarship posits EI as universally beneficial; thus, its inverse relationship with leadership among females challenges foundational assumptions and demands rigorous explanation.

From a cultural perspective, Saudi society’s ongoing transition under Vision 2030 creates a unique liminal space where traditional gender norms coexist uneasily with modernization imperatives. Female nursing students are socialized within family and community contexts that emphasize modesty, deference, and relational harmony, values that cultivate emotional intelligence but may conflict with assertive, decisive leadership behaviors. High EI in this context may actually amplify awareness of these cultural constraints, leading emotionally perceptive students to self-censor leadership aspirations to avoid social disapproval or role conflict. This phenomenon aligns with “stereotype threat” theory (48), wherein awareness of negative stereotypes about one’s group impairs performance in stereotype-relevant domains. Female students with high EI may be more acutely aware of societal skepticism toward female leadership, triggering anxiety that undermines leadership self-efficacy.

The educational system’s structure and instructor qualifications merit critical scrutiny. If nursing faculty predominantly trained in traditional pedagogical models that conflate leadership with authoritative, hierarchical styles, they may fail to recognize or validate relational, emotionally intelligent leadership approaches more common among female students. Worse, they may actively discourage such approaches as insufficiently “professional” or too “soft.” This pedagogical mismatch could explain why high EI becomes a liability: female students who prioritize empathy, collaboration, and emotional support may receive lower leadership competency ratings from evaluators who expect directive, command-oriented behaviors. The assessment instruments themselves may embed masculine leadership prototypes, systematically undervaluing communal competencies.

Additionally, instructor gender and cultural background influence evaluation bias. If male instructors or those trained abroad (common in Saudi nursing education due to reliance on international faculty) hold implicit biases about gender and leadership, they may rate female students’ leadership lower regardless of demonstrated competencies. Research on gender bias in educational settings consistently shows that women receive harsher evaluations for identical behaviors compared to men, particularly in male-typed domains like leadership (49). High-EI female students may intuitively sense these biases, leading to reduced confidence and withdrawal from leadership opportunities, a self-fulfilling prophecy wherein cultural and institutional barriers suppress potential.

From a BMF perspective, the negative EI-LC relationship may reflect informational entropy at the systemic level. The Saudi nursing education environment, influenced by international faculty, Westernized curricula, and traditional cultural norms, sends contradictory signals about leadership: “be empathetic but decisive,” “be collaborative but authoritative,” “be feminine but leader-like.” Female students with high EI, more attuned to these conflicting messages, may experience cognitive dissonance that paralyzes leadership development. The mindsponge mechanism becomes dysfunctional when environmental information is internally contradictory; instead of selective absorption, students experience rejection of both communal and agentic leadership identities, resulting in lower LC despite high emotional resources.

Finally, peer dynamics and gendered socialization within cohorts may contribute. Female students may face social sanctions from female peers for exhibiting leadership, perceived as violating in-group norms of equality and collaboration. High-EI students, more sensitive to peer relationships, may suppress leadership behaviors to maintain social harmony, prioritizing belongingness over advancement. This dynamic, documented in studies of female professional networks (50), suggests that the negative EI-LC relationship is not individual pathology but rational adaptation to structural and social constraints.

### 4.5 Contrasting with broader literature and contextualizing findings

Our findings differ from several international studies, underscoring the importance of both developmental stage and cultural context when interpreting emotional intelligence (EI) and leadership competency. Previous research has often highlighted EI as a key driver of nursing leadership; however, these studies typically focus on experienced nurses with many years of practice. For example, Akerjordet and Severinsson (51) concluded that EI plays a critical role in empowering nurse leaders, but its benefits appear more pronounced later in professional development. Recent evidence supports this view: Svetic Cisic et al. (7) found that EI significantly enhances leadership effectiveness by improving team cohesion, resilience, and decision-making, yet these advantages were most evident among nurses in formal leadership positions rather than students or novices. This suggests that EI’s positive impact on leadership may require not only emotional maturity but also organizational authority and practical experience to fully manifest.

Similarly, Miao et al. (52) meta-analysis found a strong positive relationship between EI and servant leadership (ρ̂ = 0.57), but did not account for gender differences or the moderating role of self-esteem (SE). In contrast, our study focuses on nursing students during critical developmental transitions, revealing a more nuanced picture: gendered socialization processes appear to temporarily suppress EI’s leadership benefits for females until SE is sufficiently developed.

This dynamic is particularly salient in the Saudi context, where cultural and educational factors uniquely shape leadership development. A recent cross-sectional study conducted at Al-Majmaah University found that only 8.1% of Saudi nursing students exhibited high levels of self-esteem, significantly lower than international benchmarks (53). This disparity suggests that Saudi students may face distinct challenges in developing self-worth, which could directly influence their leadership self-efficacy and professional confidence. The contrast between these findings and those from Western contexts may reflect more profound cultural divergences in conceptualizing and enacting leadership. In individualistic societies, transformational leadership styles, characterized by emotional intelligence (EI), interpersonal influence, and visionary motivation, are often celebrated and rewarded (54). Emotional intelligence in these contexts is both a predictor of leadership success and a mediator of organizational commitment and team cohesion.

Conversely, Saudi Arabia’s collectivist orientation and hierarchical social structure foster leadership models prioritizing communal harmony, loyalty, and ethical accountability over individual charisma or innovation. Leadership in Saudi organizations is often shaped by Islamic principles, tribal traditions, and consultative decision-making, emphasizing group success and moral integrity (55). These values align with collectivistic leadership approaches, which advocate for shared, team-based, and networked leadership models that distribute influence across members rather than centralizing it in a single figure (56). Such cultural variations underscore the importance of developing culturally contingent leadership development frameworks. Rather than assuming the universal applicability of Western-derived models, leadership training in Saudi Arabia should integrate indigenous values and social norms to enhance relevance and effectiveness. This approach not only respects cultural identity but also promotes leadership styles that are congruent with local expectations and organizational realities.

### 4.6 Additional support from comparative studies

These findings are further supported by Pérez-Fuentes et al. (14), who demonstrated that emotional intelligence, self-efficacy, and empathy significantly predict overall self-esteem in nursing professionals, with variations observed across years of experience. Their results affirm the critical role of self-esteem as a psychological resource in nursing development, reinforcing our conclusion that self-esteem moderates the impact of emotional intelligence on leadership competency, particularly for female students navigating identity formation during early clinical exposure. Moreover, Alshammari et al. (57) reported that emotional intelligence was positively associated with authentic leadership among Saudi nursing leaders, but did not find gender to be a significant predictor of EI, highlighting the need to explore how gender moderates the translation of EI into leadership outcomes. Our study addresses this gap by demonstrating that while gender may not predict EI levels directly, it fundamentally moderates the EI-LC relationship through differential socialization processes and information-processing mechanisms described by GITT and BMF frameworks.

### 4.7 Implications for nursing education and practice

#### 4.7.1 Theoretical implications

This study makes significant theoretical contributions by demonstrating that emotional intelligence operates not as a universal leadership enabler but as a contextually contingent resource whose efficacy depends on gender, cultural context, self-esteem, and information-processing mechanisms. By integrating social role theory, Benner’s developmental model, GITT, and BMF, we provide a comprehensive framework explaining why identical psychological resources (high EI) produce opposite leadership outcomes across genders. This challenges dominant Western-centric models that assume linear EI-leadership relationships and underscores the necessity of culturally grounded, gender-sensitive theories in nursing leadership research.

Furthermore, our findings extend the BMF by demonstrating its applicability to professional identity formation and competency development in healthcare education. The mindsponge mechanism’s role in selective information absorption based on cost–benefit evaluation offers a powerful explanatory model for understanding how cultural and institutional environments shape differential leadership trajectories. Future theoretical development should incorporate intersectional perspectives, examining how multiple identities (gender, nationality, socioeconomic status) interact with cognitive processing frameworks to produce diverse developmental pathways.

#### 4.7.2 Policy implications

At the national level, Saudi Arabia’s Vision 2030 healthcare transformation must move beyond rhetorical commitments to women’s empowerment and implement concrete policy reforms addressing systemic barriers identified in this study. These include:

1. Curriculum Reform: Mandate integration of culturally responsive, gender-inclusive leadership development modules throughout nursing programs, explicitly teaching multiple leadership models (agentic, communal, distributed, servant) and validating diverse expressions of leadership competency.
2. Faculty Development: Require comprehensive training for nursing educators in gender bias recognition, culturally sensitive pedagogy, and assessment practices that avoid privileging masculine leadership prototypes. Consider targets for hiring Saudi female nursing faculty who can serve as role models and mentors.
3. Assessment Standardization: Develop and validate leadership competency assessment tools specifically for Saudi nursing contexts that capture both agentic and communal dimensions, ensuring measurement equivalence across genders and alignment with collectivist cultural values.
4. Institutional Accountability: Establish monitoring mechanisms tracking gender disparities in leadership opportunities, clinical placements, and faculty evaluations, with transparent reporting and accountability measures for programs exhibiting persistent inequities.

#### 4.7.3 Practical implications for nursing education

These findings have significant implications for nursing education, particularly within the framework of Saudi Vision 2030.

##### 4.7.3.1 Gender-specific pathways

Nursing curricula should acknowledge distinct leadership development trajectories. For female students, integrating self-esteem (SE)-building strategies, such as reflective practice, mentorship, and simulation-based learning, can help leverage their emotional intelligence (EI) advantage. Specifically, structured mentorship programs pairing female students with established female nursing leaders can provide role models who successfully navigate cultural constraints and demonstrate that high EI can translate into leadership when supported by strong professional identity and institutional validation. High-fidelity simulation has been shown to enhance confidence and clinical decision-making among Saudi nursing students when culturally adapted (58). Simulation scenarios should explicitly incorporate leadership dilemmas requiring both emotional intelligence and decisive action, allowing female students to practice integrating these competencies in safe environments while receiving constructive feedback that validates communal leadership approaches. For male students, EI training should focus on developing communal competencies as compensatory skills. This should include structured empathy-building exercises, interpersonal communication workshops, and reflective practice emphasizing emotional awareness, areas where male students may receive less socialization but that are essential for holistic nursing leadership. This approach aligns with culturally responsive pedagogy that recognizes diverse developmental needs across gender lines.

##### 4.7.3.2 Developmental timing

Leadership interventions should be aligned with Benner’s (27) novice-to-expert model, particularly during the third-year “competent” stage when clinical responsibilities and identity tensions intensify. Scaffolded learning experiences should match developmental readiness, with early stages focusing on foundational EI skills and later stages emphasizing leadership application in clinical contexts. Given the underrepresentation of second-year students in our sample, future studies should oversample early-stage students to better understand leadership development trajectories from novice through advanced beginner stages, ensuring interventions are appropriately calibrated to developmental readiness.

##### 4.7.3.3 Reframing EI

Emotional intelligence should be reconceptualized not as a universally activating trait but as a gendered resource requiring tailored developmental mechanisms. Training modules must reflect this nuance, acknowledging that EI’s impact on leadership varies by gender and cultural context. Educators should explicitly teach students about the GITT and BMF frameworks, helping them understand how their own information processing and value systems shape leadership identity formation, thereby fostering metacognitive awareness that enables intentional development.

##### 4.7.3.4 Assessment reforms

Competency evaluations must account for gendered expressions of leadership, distinguishing between agentic versus communal styles. Inclusive, multi-dimensional frameworks are essential to avoid privileging traditionally masculine traits and to ensure equitable assessment. Batt et al. (59) emphasize that competency frameworks must be contextually grounded and inclusive to support diverse leadership expressions. Rubrics should explicitly include criteria such as “facilitates team collaboration,” “demonstrates emotional attunement,” and “builds consensus,” competencies often exhibited by high-EI female students but undervalued in traditional assessment frameworks.

##### 4.7.3.5 Professional identity and Vision 2030 integration

Targeted professional identity programs are needed to address cultural expectations and stereotyping challenges for both genders. As Almutairi and McCarthy (41) highlight, psychological empowerment must accompany structural reforms to foster leadership confidence, especially among women. These programs should help students navigate the intersection of professional identity and cultural norms, promoting self-efficacy beyond traditional gender roles. Institutional partnerships with healthcare organizations implementing Vision 2030 initiatives can provide students with exposure to emerging, more egalitarian leadership models and opportunities to observe female nurses in senior leadership positions, thereby expanding their conception of possible professional futures.

### 4.8 Future research directions

Longitudinal studies are essential to track how these gendered patterns evolve into professional practice, examining whether females’ SE-based advantage persists or is diminished by organizational structures. Such studies could follow cohorts from graduation through their early career years, providing valuable insights into how educational interventions translate into professional practice and how organizational factors mediate these relationships.

Experimental studies testing gender-specific interventions are needed for causal evidence. Randomized controlled trials could evaluate the effectiveness of targeted interventions designed to enhance self-esteem among female students and emotional intelligence among male students, providing empirical support for the proposed educational reforms.

Cross-regional studies within Saudi Arabia and international comparative research would test generalizability and cultural mediation. Comparing findings from different regions within Saudi Arabia could reveal important variations based on local cultural norms, while international comparisons could help distinguish culturally specific phenomena from more universal patterns. Particular attention should be paid to other Gulf Cooperation Council (GCC) nations undergoing similar modernization trajectories, as well as comparisons with non-Western collectivist societies (e.g., East Asian nursing education contexts) to isolate culture-specific from broader collectivist mechanisms.

Mixed-methods approaches exploring lived experiences would deepen understanding of cultural expectations shaping identity. Qualitative components could explore how students themselves make sense of their experiences and navigate the complex terrain of gender, culture, and professional identity. Focus groups and narrative interviews examining critical incidents where students felt their leadership was affirmed or rejected could illuminate the specific mechanisms through which institutional and cultural factors shape self-perception and competency development.

Neurophysiological research could illuminate cognitive mechanisms, especially the negative EI-LC correlation in females. Such research might explore the neurological underpinnings of emotional processing and decision-making in leadership contexts, potentially revealing biological factors that interact with social and cultural influences. Functional neuroimaging studies examining differences in emotional regulation and executive function activation patterns during leadership tasks could test whether high-EI females experience greater cognitive conflict (reflected in increased prefrontal activation) when reconciling empathetic impulses with assertive leadership demands, providing neurobiological evidence for the informational entropy hypothesis derived from BMF.

Additionally, intervention research should prioritize evaluation of BMF-informed pedagogical approaches that explicitly teach students to recognize and modify their information filtering mechanisms, assess whether metacognitive training enhances leadership self-efficacy, and determine optimal timing for such interventions within the developmental trajectory.

### 4.9 Study limitations

While this study provides valuable insights, several limitations warrant acknowledgment. The cross-sectional design precludes causal inference; longitudinal research is necessary to establish temporal relationships between EI, SE, and LC development. The underrepresentation of second-year students (16.9%), while reflecting natural program attrition, may limit generalizability to early developmental stages. However, sensitivity analyses excluding this cohort confirmed result robustness. The reliance on self-report measures introduces potential response bias, particularly for socially desirable constructs like leadership. Future studies should incorporate multi-source assessments (peer, faculty, clinical preceptor evaluations) to triangulate findings. The single-institution setting, while providing cultural homogeneity, limits generalizability across Saudi Arabia’s diverse regions; multi-site studies are needed. Finally, the GPA range (4-point scale) may exhibit ceiling effects (*M* = 3.96), potentially attenuating correlations with leadership variables; future research should examine broader performance ranges or alternative achievement metrics.

## 5 Conclusion

Our study advances understanding of leadership development in nursing by demonstrating that emotional intelligence (EI) and self-esteem (SE) operate through fundamentally different, gender-specific mechanisms within the Saudi Arabian context. The findings challenge universal assumptions about EI’s leadership benefits, revealing instead a complex interplay shaped by gender norms, cultural expectations under Vision 2030, and information-processing dynamics explained through Granular Interaction Thinking Theory (GITT) and Bayesian Mindsponge Framework (BMF). By integrating social role theory with Benner’s developmental model and entropy-based value formation theory (44), we provide a theoretically robust framework acknowledging gender as a critical moderator of psychological pathways to leadership.

Addressing our study aims: (1) Female nursing students demonstrate significantly higher EI but paradoxically lower leadership competency compared to males, with self-esteem emerging as the critical enabling variable for females (*β* = 2.27, *p* = 0.001) while showing inverse effects for males (*β* = −1.78, *p* < 0.001). (2) These patterns reflect informational entropy in value formation, wherein high-EI females experience cognitive conflict between communal identities and agentic leadership expectations, resulting in systematic devaluation of emotional competencies as leadership assets. Through the mindsponge mechanism, SE reduces this entropy for females, facilitating coherent integration of EI into leadership value systems, while for males, SE paradoxically diminishes compensatory motivation in a counter-stereotypical profession. (3) The significant EI × Gender interaction (*β* = −0.37, *p* = 0.020) confirms contextual contingency rather than universal applicability of EI’s leadership benefits.

## Data Availability

The datasets presented in this article are not readily available because the raw data supporting the conclusions of this article are available from the authors upon reasonable request. Requests to access the datasets should be directed to fathia.hassan@nbu.edu.sa.
